# Die Nutzbarmachung von Daten für Public Health und Gesundheitsversorgung – ein gemeinsames Ziel der EU-Mitgliedsstaaten

**DOI:** 10.1007/s00103-021-03317-w

**Published:** 2021-04-14

**Authors:** Ingo Meyer

**Affiliations:** PMV forschungsgruppe, Universität zu Köln, Medizinische Fakultät und Uniklinik Köln, Herderstr. 52, 50931 Köln, Deutschland

**Keywords:** Evidenz, Europa, Zusammenarbeit, Erfahrungsaustausch, Evidence, Europe, Collaboration, Exchange

## Abstract

Die Gesundheit der Bevölkerung – im Sinne von Prävention und Versorgung – braucht gute Daten. Daten unterstützen die Entscheidungsfindung auf der Systemebene ebenso wie konkrete Maßnahmen auf der Individualebene. Daten sind außerdem Voraussetzung für Forschung und die Entwicklung von Innovation. Gleichzeitig sind Daten Mangelware: Sie liegen nur in Ausschnitten oder für ganze Systemteile überhaupt nicht vor, sie decken Sachverhalte nur approximativ ab oder sind in Formaten gebunden, die eine automatisierte (bzw. skalierbare) Verarbeitung erschweren bis unmöglich machen. Zudem sind gerade Gesundheitsdaten höchst sensibel und entsprechend geschützt, was ihre Verwertung noch herausfordernder macht.

Mit diesen Herausforderungen steht Deutschland nicht allein da, sie finden sich in ähnlicher Form in allen anderen Mitgliedsstaaten der Europäischen Union (EU). Das Potenzial einer gemeinsamen Problemdefinition sowie der gemeinsamen Entwicklung von Lösungen wird in Deutschland bisher aber nur wenig genutzt. Der Artikel gibt einen Einblick in verschiedene EU-Initiativen rund um das Thema Gesundheitsdaten. Im Vordergrund steht dabei die Frage, wie deutsche Akteur:innen Zugang zu diesen Initiativen bekommen können, um so einerseits vom Wissen und den Erfahrungen in den Nachbarländern zu profitieren, aber auch um eigenes Wissen in diesen Kreis zurückgeben zu können.

## Abstract (en)

Population health – understood both as prevention and healthcare – needs good data. Data can support decision making on the system level as well as the delivery of services for each individual. Furthermore, data are a prerequisite for research and innovation. At the same time, health data are hard to come by: coverage is spotty or – for certain parts of the system – lacking, available data only provides a proxy view of actual prevention or healthcare, or it exists in formats that hamper or prevent automated processing at scale. Furthermore, the high sensitivity of health data necessitates an equally high degree of protection, further complicating access.

These challenges are not specific to Germany but are common in all member states of the European Union. However, up to now, German stakeholders tend to neglect the potential of a joint European approach to these challenges. The article provides an overview of different European initiatives in the domain of health data. The focus is on how German stakeholders can gain better access to these initiatives to benefit from the knowledge and experiences of neighbouring countries, but also to feed back their own knowledge into the European loop.

## Daten – mehr als das neue Öl

„The world’s most valuable resource is no longer oil, but data“, titelte *The Economist* im Jahr 2017 [1]. Dieses geflügelte Wort ist schon seit Längerem in aller Munde. 2015 bezeichnete Bundeskanzlerin Angela Merkel Daten als die „Rohstoffe des 21. Jahrhunderts“, zu der Zeit im Kontext der europäischen Verhandlungen über die Datenschutz-Grundverordnung [2]. Das Konzept wird aber auch immer wieder kritisiert, einerseits wegen der inhärenten und schwierigen Abwägung zwischen wirtschaftlichen Interessen und dem Schutz von Bürgerrechten, andererseits auch wegen konzeptioneller Fallstricke. Rusche [3] sieht Lücken in der Analogie: Anders als Öl würden Daten bei Verwendung nicht verbraucht, sondern könnten mehrfach verwendet werden. Außerdem entstehen Daten in vielen Organisationen als Nebenprodukt und müssten entsprechend nicht zugekauft, sondern – mit seinen Worten – „veredelt“ werden.

Im Gesundheitswesen – Public Health, Versorgung, Forschung – ist diese Debatte schon viel älter. Ob evidenzbasierte Medizin auf der Ebene individueller Versorgung [4] oder evidenzbasierte gesundheitspolitische Entscheidungsfindung auf der Ebene der Bevölkerung [5]: Die Verwendung von Evidenz – im Sinne verarbeiteter Daten – ist heute essenzieller Bestandteil der Schaffung und Erhaltung von Gesundheit. Trotzdem entsteht der Eindruck, dass die großen Fortschritte in der Datenverarbeitung, die in den letzten Jahren in der Industrie, dem Onlinehandel oder den sozialen Medien zu beobachten sind, nur mit großer Verzögerung oder gar nicht ihren Weg ins Gesundheitswesen finden. Ein wichtiger Grund ist sicher der Datenschutz, der fast nirgendwo wichtiger ist als hier, wo die verarbeiteten Daten nahezu immer höchste Sensibilität haben.

Man kann sagen, dass die Nutzbarmachung und Verwendung von Gesundheitsdaten noch stärker im Zeichen der Kosten-Nutzen-Abwägung stehen, als dies in anderen Bereichen (vielleicht mit Ausnahme des Sicherheitssektors) der Fall ist. Auf der Nutzenseite steht die Unterstützungsleistung von Daten. Innovation in Prävention und Versorgung, Entscheidungen von Gesundheitsbehörden, Regierungsstellen und Politiker:innen, die Arbeit von Aufsichtsbehörden, die Erbringung individueller Versorgungsleistungen, die Durchführung von Public-Health-Maßnahmen bis hin zur Evaluation und Forschung: Alle diese Bereiche profitieren von Daten oder sind ohne Daten praktisch handlungsunfähig. Auf der Seite der Kosten stehen an erster Stelle die Risiken, die von unzureichendem Schutz der Daten ausgehen. Hinzukommen aber auch technische Barrieren (vom Faxgerät bis zu fehlender semantischer Interoperabilität^1^), der Bedarf für finanzielle Investition, der hohe Zeitaufwand für das notwendige Changemanagement, der häufige Widerspruch zwischen prinzipiell Möglichem und tatsächlich Benötigtem, aber auch ganz konkrete Fragen der Qualitätssicherung von Daten und Ergebnissen sowie der Entwicklung und Etablierung geeigneter Methoden.

Die oben skizzierten Herausforderungen bei der Nutzung von Gesundheitsdaten sind nicht auf Deutschland beschränkt, sondern globaler Natur. Ihre genaue Ausprägung variiert zwar mit den Rahmenbedingungen der einzelnen Staaten oder globalen Regionen, aber prinzipiell finden sich Diskussionen, Initiativen, Projekte und Lösungen auf dem gesamten Globus. Dies bedeutet, dass die Suche nach Lösungen für deutsche Probleme nicht nur in Deutschland stattfinden muss. Wissen, Gelerntes und gute Beispiele aus anderen Ländern können herangezogen werden, um beispielsweise das Wiederholen von anderswo gemachten Fehlern zu vermeiden. Aus deutscher Perspektive ist die Europäische Union nicht nur die räumlich nächste supranationale Einheit für einen solchen wechselseitigen Lernprozess. Mit dem geteilten Wirtschaftsraum und dem in entscheidenden Bereichen angeglichenen Rechtsrahmen herrschen auch gute Rahmenbedingungen für den Transfer von Erfahrungen. Gleichzeitig sind das Subsidiaritätsprinzip und die Souveränität der Mitgliedsstaaten so tief verankert, dass viel Spielraum für die Beachtung nationaler und auch regionaler Besonderheiten besteht.

Vor diesem Hintergrund zielt dieser Beitrag darauf ab, das bisher ungenutzte Potenzial europäischer Kooperation ebenso aufzuzeigen wie konkrete Wege für deutsche Akteur:innen, dieses Potenzial nutzbar zu machen. Der Beitrag liefert dazu zunächst einen Überblick über europäische Initiativen mit Bezug zu Gesundheitsdaten. Dabei werden sowohl Initiativen der hochrangigen Politik im Sinne von Europäischer Kommission, Europäischem Rat und den jeweiligen Ratspräsidentschaften dargestellt als auch Initiativen mit Bottom-up-Charakter („von unten nach oben“). In der Diskussion geht es um die Frage, wie deutsche Akteure besser als bisher Zugang zu der europäischen „Wissens- und Erfahrungsdatenbank“ erlangen können, die diese beschriebenen Initiativen darstellen. Das Fazit ist schließlich ein Appell, die dargestellten Möglichkeiten einer stärkeren europäischen Zusammenarbeit zum Thema Gesundheitsdaten zeitnah zu nutzen.

## Europäische Initiativen mit Bezug zu Gesundheitsdaten

Europäische Initiativen mit Bezug zu Gesundheitsdaten lassen sich in 2 Kategorien fassen. Auf der einen Seite stehen Initiativen der hochrangigen Politik, insbesondere der Europäischen Kommission und des Europäischen Rates respektive der jeweiligen Ratspräsidentschaft. Diese geben vor allen Dingen den Rahmen vor und wirken sich so beispielsweise auf die Verplanung europäischer Forschungsfördermittel in Programmen wie Horizon 2020 oder Horizon Europe aus, aber auch auf europäische Gesetzgebungsverfahren. Auf der anderen Seite steht eine ungleich größere Zahl von Initiativen, die – im Vergleich zur hochrangigen Politik – eher durch einen Bottom-up-Charakter oder zumindest durch eine größere Offenheit und Zugänglichkeit gekennzeichnet sind: Akteursnetzwerke, kooperative Forschungsprojekte oder auch gezielte Einzelmaßnahmen. Diese sind meist deutlich konkreter, was die bearbeiteten Themen angeht, folgen aber in der Regel den „von oben“ gegebenen politischen Leitlinien, nicht zuletzt da aus deren Kontext häufig die Finanzierung erfolgt.

### In der europäischen Politik

In der hochrangigen europäischen Politik spielt das Thema Daten, und speziell auch Gesundheitsdaten, seit Langem eine wichtige Rolle. Aktuell sind hier zum Beispiel der digitale Binnenmarkt [6] und die europäische Datenstrategie [7] zu nennen.

Die europäische Datenstrategie benennt explizit die Bedeutung von Daten für eine bessere Gesundheitsversorgung. Sie bemängelt, dass sensible Daten aus öffentlichen Datenbanken (wie beispielsweise des Gesundheitswesens) häufig nicht für Forschungszwecke zur Verfügung gestellt werden, da es weder Kapazitäten noch Konzepte gibt, solche Forschungen in Übereinstimmung mit den Vorschriften des Datenschutzes durchzuführen. Gefordert wird hierfür die Einrichtung sogenannter europäischer Datenräume, einschließlich eines Gesundheitsdatenraumes. Dieser soll für Fortschritte bei der Prävention, Erkennung und Heilung von Krankheiten genutzt werden ebenso für evidenzbasierte Entscheidungen zur Verbesserung der Zugänglichkeit, Wirksamkeit und Nachhaltigkeit der Gesundheitssysteme.

Die Forderungen hinsichtlich der Gesundheitsdaten gehen wesentlich auf eine Kommunikation aus dem Jahr 2018 zurück [8] und spielen auch im Rahmen der deutschen EU-Ratspräsidentschaft 2020 eine wichtige Rolle. Im Rahmen der hochrangigen Konferenz „Digital Health 2020 – EU on the Move“ bekräftigen sowohl Bundesgesundheitsminister Jens Spahn als auch die Kommissarin für Gesundheit und Lebensmittelsicherheit, Stella Kyriakides, und der Kommissar für den Binnenmarkt, Thierry Breton, die Notwendigkeit eines länderübergreifenden Rahmens für den Gesundheitsdatenaustausch [9, 10].

### Bottom-up-Initiativen

Wie oben schon gesagt ist die Zielsetzung hier eine andere als im Bereich der hohen Politik und es geht eher um konkrete Probleme und Fragestellungen. Der Versuch, die europäische Landschaft solcher Bottom-up-Initiativen mit einem Anspruch auf Vollständigkeit zu erfassen und zu strukturieren, wäre ein eigenes Forschungsprojekt und liegt – leider – außerhalb der Möglichkeiten eines solchen Beitrags. Möglich ist aber eine Beschreibung zweier großer Zugangswege zu europäischer Vernetzung: einerseits über bestehende Akteursnetzwerke zu unterschiedlichen Themen, andererseits über mit EU-Mitteln geförderte Forschungsprojekte.

#### Akteursnetzwerke

Der Begriff „Akteursnetzwerk“ steht hier als Oberbegriff für eine Vielzahl von Netzwerken, Plattformen, Foren und Arbeitsgruppen unterschiedlicher formaler Verfasstheit und Zielsetzung. Gemeinsam ist allen, dass individuelle und kollektive Akteur:innen dort zusammenkommen, um Themen zu bearbeiten.

Eine Einstiegsmöglichkeit in diese Welt der Netzwerke bietet die *EU Health Policy Platform* [11], eine Onlineplattform für den Austausch zwischen Europäischer Kommission und Akteur:innen aus dem Gesundheitsbereich. Die Plattform bietet nicht nur Zugang zu diesen Akteur:innen, sondern auch zu einer größeren Zahl thematischer Netze. Der Schwerpunkt liegt hier überwiegend auf Gesundheitsthemen im Allgemeinen, wobei Gesundheitsdaten oft als Unterthema behandelt werden. Eine ähnliche Konstellation findet sich in der *European Innovation Partnership on Active and Healthy Ageing* (EIP on AHA, [12]). Anders als der Name nahelegt, befasst sich die Initiative schon seit Längerem nicht mehr ausschließlich mit der alternden Gesellschaft, sondern mit Prävention und Versorgung im Allgemeinen. Verschiedene Arbeitsgruppen beschäftigen sich u. a. mit Medikationsadhärenz und integrierter Versorgung, wobei die Nutzung von Gesundheitsdaten als Querschnittsthema mit bearbeitet wird [13].

Ein weiteres europäisches Netzwerk ist beispielsweise *EHTEL – European Health Telematics Association* [14], die einen Schwerpunkt auf E‑Health und Gesundheitstelematik legt und aktuell auch Themen wie den europäischen Gesundheitsdatenraum, datengetriebene Versorgungsintegration und Analysemethoden wie künstliche Intelligenz bearbeitet. Das *European Institute for Innovation through Health Data* [15] hat seinen Ursprung in europäischen Verbundprojekten zur Nutzbarmachung elektronischer Gesundheitsakten und zur semantischen Interoperabilität und hat weiterhin einen Schwerpunkt in Aspekten der stationären Versorgung. Die *European Connected Health Alliance *(ECHAlliance; [16]) ist als europäisches Netzwerk gestartet und vereint nach eigenen Angaben mittlerweile Mitglieder aus 78 Ländern weltweit. Im Vordergrund steht auch die Vernetzung rund um das Thema digitale Gesundheit. Die ECHAlliance ist in sogenannten regionalen Ökosystemen organisiert, von denen sich auch 3 in Deutschland finden: im Rheinland, in Hessen und der Metropolregion Nürnberg. Die Personal Connected Health Alliance (PCHAlliance; [17]) kann als Industrieverband verstanden werden, der 2006 unter dem Namen Continua Health Alliance gegründet wurde und heute zur US-amerikanischen Healthcare Information and Management Systems Society (HIMSS) gehört. Der Schwerpunkt der Arbeit der PCHAlliance liegt auf der Entwicklung von Interoperabilitätsstandards. In diesem Zusammenhang lassen sich auch die internationalen Verbände Health Level 7 (HL7; [18]) und Systematized Nomenclature of Medicine (SNOMED; [19]) nennen, die sich ebenfalls mit der Entwicklung und Verbreitung von Interoperabilitätsstandards bzw. Terminologien beschäftigen.

#### Verbundprojekte

Eine weitere Zugangsmöglichkeit sind mit EU-Mitteln geförderte Verbundprojekte, die Organisationen aus allen EU-Mitgliedstaaten und einer Reihe assoziierter Staaten (unter anderem die Schweiz, die USA, Kanada und Japan) offenstehen. Die Fördermittel werden über die großen Rahmenprogramme für Forschung und Innovation (Horizon 2020 [20] für den Zeitraum 2014 bis 2020, Horizon Europe [21] für 2021–2027), den Regionalentwicklungs- [22] sowie Sozialfonds [23] und über spezielle Programme (wie die *innovative medicines inititiative – imi* [24]) vergeben. Das Thema Gesundheitsdaten ist dabei sowohl explizit (gerade bei Horizon und *imi*) als auch implizit (z. B. in der Regionalentwicklung) Gegenstand der zeitlich befristeten Arbeitsprogramme, die den inhaltlichen Rahmen für die Mittelvergabe vorgeben.

## Zugangswege für deutsche Akteur:innen

Wie kann der Zugang zu den gerade beschriebenen Initiativen für deutsche Akteur:innen konkret aussehen? Wie lässt sich einerseits vom Wissen und den Erfahrungen zum Thema Gesundheitsdaten in den Nachbarländern profitieren und andererseits eigenes Wissen und eigene Positionen in den europäischen Raum zurückgeben?

Den Zugang zum Austausch auf der Ebene der europäischen Politik dürften sich unmittelbar insbesondere Vertreter:innen von Regierungseinrichtungen (Bundes- oder Landesministerien, aber auch Bundesoberbehörden), Bundesverbänden und großen Organisationen verschaffen können. Dies kann einerseits direkt über die europäischen Institutionen erfolgen (z. B. über die entsprechenden Generaldirektionen der Europäischen Kommission oder über das EU-Parlament) oder auf dem Weg über die Bundesregierung. Das starke Engagement der Regierung für das Thema Gesundheitsdaten während der vergangenen Ratspräsidentschaft hat hierfür sicherlich einen guten Nährboden bereitet. Die Möglichkeiten auf dieser Ebene bestehen vor allen Dingen in der Schaffung und Verbesserung von Rahmenbedingungen, sei es in der Gesetzgebung oder in der Finanzierung (insb. von Forschung und Innovation).

Die Bottom-up-Initiativen sind hingegen eher an konkreten Problemen und Fragestellungen orientiert und bieten somit einen unmittelbaren Nutzen, z. B. im Sinne von umsetzbaren Konzepten, finanzieller Förderung oder auch gemeinsamen Publikationen. In Verbundprojekten können mit finanzieller Unterstützung eigene Ideen entwickelt, getestet und implementiert werden, während man gleichzeitig von den Erfahrungen anderer profitiert. Ein Projektkonsortium mit Partnern aus mehreren Mitgliedsstaaten und Beiratsmitgliedern aus der ganzen Welt stellt schon für sich genommen ein Forum für wechselseitiges Lernen und Ideenaustausch dar. Allerdings ist der Zugang zu diesen Projekten nicht leicht. Auf der einen Seite stehen komplexe Vorgaben beispielsweise hinsichtlich der Antragsverfahren und des Berichtswesens im Projekt. Auf der anderen Seite hat der Wettbewerb um EU-Fördermittel – insbesondere in den großen Rahmenprogrammen – in den letzten Jahren deutlich zugenommen. Ausschreibungen zu einzelnen Themen mit über 100 Einreichungen im Vergleich zu 3–5 geförderten Projekten sind keine Seltenheit.

Im Vergleich dazu zeichnen sich die Netzwerke dadurch aus, dass sie meist ohne größere Hürden struktureller oder finanzieller Art zugänglich sind. Sie bieten also eine niedrigschwellige Zugangsmöglichkeit zu den laufenden Diskussionen rund um das Thema Gesundheitsdaten auf der EU-Ebene, aber auch in den einzelnen Mitgliedsstaaten. Über Konsultationen, Positionspapiere oder auch direkte Kontakte findet außerdem ein Rückfluss von Ideen und Positionen in den hochrangigen politischen Rahmen, insbesondere in die Entscheidungsprozesse der Europäischen Kommission, statt. Ein Beispiel hierfür sind die Konsultationen zu den Arbeitsprogrammen der beschriebenen Rahmenprogramme, über die sich in einem gewissen Umfang eigene Themen in der europäischen Innovationsförderung platzieren lassen. Mit Blick auf die Verbundprojekte erfüllen die Netzwerke außerdem eine weitere Funktion als kreatives Umfeld, in dem gemeinsame Projektideen und -anträge entstehen. Strategisch gesehen ist es daher sinnvoll, sich zunächst in Richtung der Netzwerke zu orientieren und über diese dann Zugang zu den Verbundprojekten zu suchen, da sich hier Menschen und Organisationen mit Antragserfahrung und mit detaillierteren Kenntnissen der aktuellen inhaltlichen Diskussion auf der EU-Ebene finden.

### Data Matters – Ein Beispiel für EU-finanzierten Erfahrungsaustausch

Abschließend soll anhand eines Beispiels sowohl ein Eindruck vom greifbaren Nutzen europäischer Kooperation vermittelt als auch gezeigt werden, wie eine eigene Zugangsstrategie (hier für Partnerorganisationen aus drei EU-Ländern) aussehen kann. Das Beispiel ist ein EU-finanziertes Projekt zum Erfahrungsaustausch zwischen europäischen Regionen aus den Jahren 2019 und 2020. Inhaltlich ging es um die bessere Nutzung von Gesundheitsdaten für politische Entscheidungsfindung, Präventions- und Versorgungsplanung sowie die datengestützte Verbesserung von Programmen und Gesundheitsdiensten.

Mit der Initiative „Digital Health Europe“ [25] hat die Europäische Kommission im Zeitraum 2019 und 2020 bereits zum zweiten Mal den Erfahrungsaustausch und den Transfer von Lösungen im Bereich digitale Gesundheitsversorgung unterstützt. Die Initiative hat hierzu unter anderem rund 45 sogenannte Twinnings organisiert und finanziert. Im Rahmen dieser Twinnings trafen Regionen mit vorhandenen Ideen, Konzepten, Produkten oder Dienstleistungen zur digitalen Versorgung auf „Zwillingsregionen“, die diese Innovationen im eigenen Kontext umsetzen wollten. Finanziert wurden dabei sowohl gemeinsame Treffen und Reisetätigkeit als auch Lizenzgebühren oder Kosten für externe Dienstleister.

Eine der geförderten Maßnahmen war ein Erfahrungsaustausch zwischen 3 europäischen Partnern zum Thema Gesundheitsdaten. Unter dem Titel „Data matters – better data to advance research, disease prevention and personalised health and care“ beteiligten sich Organisationen aus Estland, Spanien und Deutschland, um gegenseitig von Lösungen und Erfahrungen in den Bereichen 1) Dateninfrastruktur, 2) Datenintegration und -Linkage sowie 3) Datenanalyse unter Anwendung von maschinellem Lernen und künstlicher Intelligenz zu lernen.

Das estnische Sozialministerium stellte dabei für das erste Thema die nationalen Bestrebungen für eine Dateninfrastruktur vor, mit deren Hilfe eine bessere Integration verschiedener Sektoren des Gesundheits‑, Sozial- und Bildungssystems erreicht werden soll. Eine zentrale Rolle spielt hierbei die estnische E‑Government-Infrastruktur X‑Road [26], die den Datenaustausch zwischen Einrichtungen des öffentlichen und des privaten Sektors ebenso ermöglicht wie die Anbindung von Nutzerinterfaces für Bürger:innen, Unternehmen und öffentliche Bedienstete. Dabei funktioniert X‑Road als eine Art Middleware (anwendungsneutrale Programme zur Kommunikation zwischen verschiedenen Anwendungen), die insbesondere die Sicherheit der Kommunikationswege, die Authentisierung der Nutzer:innen und die Protokollierung von Datenzugriffen übernimmt. X‑Road, seine bestehenden Anwendungsfälle und die Verwendung für eine stärkere Integration von Gesundheits- und Sozialdaten wurden dabei den Twinning-Teilnehmer:innen im Rahmen eines zweitägigen Treffens in Tallinn vorgestellt. Ein weiteres Ergebnis dieses Treffens war eine Auflistung wesentlicher regulativer, finanzieller und technischer Barrieren für bessere Datenintegration in Estland, Deutschland und Spanien. Diese wies viele Gemeinsamkeiten zwischen den Ländern auf (beispielsweise hinsichtlich der negativen Folgen unterschiedlicher Auslegungen der EU-Datenschutzrichtlinie), aber auch einige Unterschiede (insbesondere hinsichtlich des Vertrauens von Bürger:innen in staatliche Stellen, das in Spanien und Estland höher zu sein scheint als in Deutschland).

Aus Deutschland beteiligte sich für das zweite Thema das Kölner Netzwerk CoRe-Net [27] mit seiner Forschungsdatenbank aus Routinedaten von 4 beteiligten gesetzlichen Krankenversicherungen, Primärdaten verschiedener Studien sowie Strukturdaten [28]. Diese Datenbank wurde in den Jahren 2018 bis 2020 in der ersten Phase des vom Bundesministerium für Bildung und Forschung (BMBF) geförderten Netzwerks erstellt und steht seit diesem Jahr zur Verfügung. Die Nutzung der Daten erfolgt zunächst durch 2 Projekte, die Teil von CoRe-Net sind und sich mit der Versorgung im letzten Lebensjahr sowie mit psychischen Komorbiditäten bei koronaren Herzerkrankungen befassen und diese für den Kölner Kontext erforschen. Darüber hinaus steht die CoRe-Net-Datenbank auf Antrag für weitere Forschungsprojekte, Auswertungen zu einzelnen Forschungsfragen und andere Anwendungsszenarien wie kommunale Versorgungsberichte zur Verfügung. Das Twinning-Treffen in Köln befasste sich darüber hinaus mit der Entwicklung eines Logikmodells zur Herleitung von Effekten einer stärker integrierten Gesundheits- und Sozialversorgung, das in allen 3 nationalen Kontexten gleichermaßen zum Einsatz kommen kann.

Der spanische Projektpartner für das dritte Thema war der kommunale Gesundheitsversorger der katalanischen Stadt Badalona, BSA – Badalona Serveis Assistencials, der sein Data Lab für die Analyse komplexer und großvolumiger Gesundheitsdatensätze ebenso vorstellte wie eine Reihe von Forschungsprojekten, die in diesem Data Lab durchgeführt wurden. Über das Data Lab führt BSA Daten aus verschiedenen, heterogenen Quellen zusammen, die elektronische Patientenakten, Routine- bzw. Abrechnungsdaten, aber auch Ärzt:innenbriefe im Freitext und Daten aus von Bürger:innen genutzten Gesundheits-Apps umfassen. Diese Daten werden zusammengeführt, für die Auswertung vorbereitet und dann mit Methoden des maschinellen Lernens und der künstlichen Intelligenz ausgewertet. Anwendungsfälle sind Auswertungen zu nicht wahrgenommenen ambulanten Terminen, zur effizienteren Nutzung von Operationsräumen oder zum Einsatz von Fallmanager:innen in der Versorgung von Schlaganfallpatient:innen. Wegen der Coronapandemie musste ein ursprünglich geplanter Besuch der Twinning-Partner in Badalona ausfallen und wurde durch ein virtuelles Treffen ersetzt.

Neben den 3 Treffen zu den thematischen Schwerpunkten fanden zu deren Vor- und Nachbereitung außerdem mehrere Videokonferenzen statt. Insgesamt beteiligten sich mehr als 50 Personen an diesem Austausch, der insgesamt 14 Monate dauerte. Nach Abschluss der Initiative einigten sich die beteiligten Akteure darauf, die in vielen Punkten lediglich begonnene Arbeit (beispielsweise am oben beschriebenen Logikmodell) sowie den inhaltlichen Austausch nach Ende der EU-Förderung mit eigenen Mitteln fortzuführen. Hierzu wird im Jahr 2021 eine Arbeitsgruppe (Special Interest Group) „Data Matters“ im Rahmen der International Foundation for Integrated Care (IFIC) eingerichtet. Diese Gruppe steht dann auch weiteren Teilnehmer:innen mit Interesse am Thema offen. Mittelfristig sollen aus dieser Gruppe heraus entsprechende Projektanträge für europäische Förderprogramme entwickelt werden.

## Fazit: Anknüpfungspunkte suchen

Der Beitrag konnte hoffentlich zeigen, wie groß einerseits das Potenzial für einen im Vergleich zu heute viel breiteren Austausch mit europäischen Partnern ist, andererseits aber auch, welche unterschiedlichen Zugangsmöglichkeiten zu einem solchen Erfahrungsaustausch für deutsche Akteur:innen bestehen und welcher konkrete Nutzen daraus ableitbar ist. Dabei versteht sich dieser Beitrag bewusst als Appell an alle Leser:innen, von diesen Möglichkeiten Gebrauch zu machen, über den (deutschen) Tellerrand zu blicken und – mit hoher Wahrscheinlichkeit – davon zu profitieren.

Dafür lassen sich Anknüpfungspunkte in 2 Richtungen suchen: zunächst natürlich mit Akteur:innen in anderen EU-Mitgliedsstaaten, die – wie gezeigt – vor ähnlichen Herausforderungen stehen, wie sie sich derzeit hier in Deutschland ergeben, und ggf. aber schon ein oder zwei Schritte weiter auf dem Weg zu einer Lösung sind, zum anderen aber vielleicht auch innerdeutsch zwischen der Public-Health-Community und der Versorgungscommunity. Denn auch hier gibt es viele Gemeinsamkeiten, was den Bedarf an guten, umfassenden und hochverfügbaren Gesundheitsdaten angeht. Diese Gemeinsamkeiten scheinen sich allerdings noch nicht in ausreichendem Maße in einer gemeinsamen Vorgehensweise niederzuschlagen.

Dass beide Anknüpfungen auch in Deutschland möglich sind, zeigen – wieder ohne Anspruch auf Vollständigkeit – Initiativen wie die Nationale Forschungsdateninfrastruktur für personenbezogene Gesundheitsdaten (NFDI4Health) [29] oder das schon beschriebene Netzwerk CoRe-Net. Ein wesentliches Ziel von NFDI4Health ist die Zusammenführung von Daten der klinischen Forschung, Epidemiologie und Public Health. Dabei hat die Initiative gerade im Bereich der Standardisierung und Interoperabilität eine starke Anbindung an europäische und internationale Gruppen/Organisationen wie HL7 und CEN/CENELEC (Europäisches Komitee für Normung/Europäisches Komitee für elektrotechnische Normung). Die Daten der CoRe-Net-Datenbank werden in der aktuell zweiten Förderphase des Projektes nicht nur für Forschungszwecke, sondern für die Erstellung von thematischen Versorgungsberichten genutzt, die zur Unterstützung der kommunalen Versorgungsplanung gedacht sind. Über die Arbeitsgruppe „Data Matters“ ist auch diese Arbeit auf europäischer Ebene vernetzt.

Im Sinne der ohnehin schon herausragenden Rolle von Evidenz im Gesundheitswesen wäre es wünschenswert, wenn diese Beispiele weitere Nachahmer:innen fänden.

## Footnotes

1 Fähigkeit zum Zusammenspiel technischer Systeme auf der Ebene der Informationseinheiten.
